# Cardiopulmonary Exercise Testing and Exercise Stress Echocardiography in Degenerative Mitral Regurgitation: An Exploratory Analysis of Functional and Left Ventricular Outcomes After Surgery

**DOI:** 10.3390/jcdd13080383

**Published:** 2026-08-12

**Authors:** Laura Besola, Giulia Bacci, Cinzia Anna Maria Papappicco, Dario Gregori, Giosuè Salvatore Falcetta, Federico Giorgi, Nicola Riccardo Pugliese, Andrea Colli

**Affiliations:** 1 Cardiac Surgery Unit, Department of Surgical, Medical and Molecular Pathology and Critical Care Medicine, University of Pisa, 56124 Pisa, Italy; laura.besola@unipi.it (L.B.);; 2 Unit of Biostatistics, Epidemiology and Public Health, Department of Cardiac, Thoracic, Vascular Sciences and Public Health, University of Padova, 35131 Padova, Italy; cinziaannamaria.papappicco@ubep.unipd.it (C.A.M.P.); dario.gregori@ubep.unipd.it (D.G.); 3 Heart Failure Laboratory, Department of Clinical and Experimental Medicine, University of Pisa, 56124 Pisa, Italy

**Keywords:** degenerative Mitral Regurgitation, left ventricular ejection fraction, cardio-pulmonary exercise test, exercise stress echocardiography, functional capacity

## Abstract

Background: Surgical correction of severe degenerative Mitral Regurgitation (DMR) is indicated in the presence of symptoms and signs of left ventricular (LV) dysfunction. However, conventional clinical and resting echocardiographic parameters may underestimate myocardial impairment. Cardiopulmonary exercise testing (CPET) and exercise stress echocardiography (ESE) may provide a more complete functional and haemodynamic assessment. Objectives: To characterize preoperative CPET and ESE findings in patients with severe DMR and to explore, in a hypothesis-generating analysis, their association with early functional and left ventricular outcomes after surgery. Methods: We included patients with severe DMR who underwent CPET and ESE before surgery. Functional improvement was defined as an improvement of at least one NYHA class from the preoperative baseline to early follow-up. LV function was evaluated as the continuous change in LVEF from discharge to early follow-up (Delta LVEF = follow-up LVEF − discharge LVEF). Baseline parameters were compared between patients who did and did not improve functionally. Random forest analyses were used only as an exploratory secondary analysis to assess whether baseline variables showed any non-linear signal of association with the outcomes and to document the instability of variable-importance rankings. Results and Conclusions: We included 60 patients, of whom 72% were in NYHA class II and all had preserved LV function. At CPET, rest and peak oxygen uptake were 6 +/− 1.5 and 17.2 +/− 5 mL/kg/min, respectively; the VE/VCO_2_ slope was 33 +/− 8.9 and the Oxygen Uptake Efficiency Slope was 1.6 (1.3–1.9). At ESE, LVOT-VTI increased from 18.3 +/− 3.9 cm at rest to 24.5 +/− 4.9 cm at peak; stroke volume from 51.0 (43.0–60.0) mL to 72.5 +/− 21.6 mL; and cardiac output from 3.8 (3.2–5.1) L/min to 9.0 +/− 3.2 L/min. No baseline clinical, CPET or ESE parameter significantly distinguished patients who improved functionally from those who did not, and early Delta LVEF was negligible. Random forest analyses did not discriminate above chance and variable-importance rankings were unstable. Patients with severe DMR and preserved LVEF showed objectively abnormal exercise responses despite mild symptoms, supporting the use of CPET and ESE for preoperative functional and haemodynamic characterization; larger prospective studies are required before any predictive or decision-support use can be proposed.

## 1. Introduction

Primary degenerative Mitral Regurgitation (DMR) is diagnosed in about 10% of Western Countries [1]. When not treated, it is associated with poor survival and quality of life. The 2020 American College of Cardiology/American Heart Association [2] and 2025 European Society of Cardiology/European Association of Cardio-Thoracic Surgery [3] guidelines give Class I indication for DMR in patients with severe regurgitation only in presence of symptoms and/or signs of left ventricle (LV) function impairment as lowering of Left Ventricle Ejection Fraction (LVEF) less to 60% and/or left ventricle end-systolic diameter (LVESD) ≥ 40 or 45 mm. However, these conventional echocardiographic parameters may not always detect myocardial dysfunction, as demonstrated by previous research [4,5,6], and the presence of symptoms is a condition that is sometimes unreliable in this subset of patients. Despite the evident benefits deriving from surgical correction, the right timing of treatment is still debatable. Considering the current published studies showing that PMR is also associated with increased mortality in those patients with preserved LV function and low comorbidity [7,8,9,10,11], we hypothesize that a complete multimodal approach, including functional assessment with cardio-pulmonary exercise testing (CPET) and exercise-stress echocardiography (ESE), is needed with these patients.

In this retrospective, exploratory, hypothesis-generating study, we characterize preoperative CPET and ESE findings in patients with severe DMR and preserved LVEF, and explore their association with early functional status and LV function after surgery.

## 2. Materials and Methods

This is a retrospective observational single-center study. We included all patients with severe DMR who underwent surgery, either repair or replacement, at the cardiac surgery unit at The University of Pisa Hospital between October 2020 and April 2024. Presence of mitral stenosis, more than mild aortic regurgitation or stenosis, previous cardiac surgery, and contraindications for CPET and ESE were exclusion criteria. Patients who did not undergo full-protocol CPET or with suboptimal ESE were excluded.

Baseline clinical (standard EuroSCORE II risk factors) and 2D transthoracic echocardiography (2D-TTE) data were collected, including conventional quantitative parameters for assessing MR severity and LV function according to the American Society of Echocardiography (ASE) guidelines [12]. Before surgery, all patients underwent CPET and ESE using standardized protocols. A graded ramp bicycle CPET was performed using a tilting, dedicated, microprocessor-controlled stress echocardiography cycle ergometer (Ergoline ergoselect 2000 GmbH, Bitz, Germany) according to a modified Bruce protocol with respiratory gas-exchange evaluation at baseline, peak exercise and recovery. The expected peak oxygen consumption (VO_2_) was calculated on the base of patient age, height, weight, and clinical history. Thw work rate increment required to reach the patient’s estimated peak VO_2_ in 8–12 min was also calculated. Breath-by-breath minute ventilation, carbon dioxide production (VCO_2_), and VO_2_ were measured, peak VO_2_ was the highest averaged 30 s VO_2_ during exercise while the anaerobic threshold (AT) was determined manually using the V-slope, ventilatory equivalents, and end-tidal partial-pressure methods. ESE was performed during the stress protocol at four stages: rest, low-load effort, peak stress and recovery. Operative and postoperative outcomes were assessed according to Mitral Valve Academic Research Consortium (MVARC) indications. Standard comprehensive 2D-TTE was performed before hospital discharge, evaluating the same conventional parameters measured at baseline. Clinical and 2D-TTE follow-up were performed after surgery; the early follow-up visit, performed at a median of 6.4 months (IQR 5.9–6.8) after discharge, was used for the present analysis.

We evaluated two outcomes at early follow-up:

(1) Improvement of functional status, defined as an improvement of at least one NYHA class from the preoperative baseline to early follow-up.

(2) Change in LV function, defined as the continuous change in LVEF between discharge and early follow-up (Delta LVEF = follow-up LVEF − discharge LVEF).

Survival at thirty days and at follow-up was not considered an outcome for the purpose of the present study.

Continuous variables are summarized as median (first-third quartile) or mean +/− standard deviation according to their distribution, and categorical variables as count (percentage). Continuous variables were compared using the Mann–Whitney U test, and categorical variables using Fisher’s exact test. The between-procedure comparison of 30-day mortality used Fisher’s exact test. Values outside pre-specified physiologically plausible ranges were treated as missing.

Both outcomes were cross-sectional clinical states ascertained at the follow-up visit; time-to-event analysis was not an endpoint of this study, and no survival probabilities were estimated. NYHA-class improvement was analyzed as a binary outcome, whereas Delta LVEF was analyzed as a continuous outcome. No data-driven or stepwise variable selection was performed. Candidate predictors were grouped a priori into clinical, resting echocardiographic, and CPET/ESE domains. Given the sample size (60 operated patients; 39 and 32 patients evaluable for the NYHA and LVEF outcomes, respectively), the number of events per candidate predictor (approximately 1.6–2.1) was well below the minimum required to develop and validate a multivariable clinical prediction model [13,14]. Random forests were therefore used only as an exploratory secondary analysis to assess whether non-linear combinations of baseline variables showed any signal of association with the outcomes and to evaluate the stability of variable-importance rankings. No split into training and testing sets was performed, because this would have produced highly unstable estimates. Internal performance was assessed using out-of-bag estimation and bootstrap resampling. Discrimination for the NYHA outcome was evaluated by the area under the ROC curve, whereas performance for Delta LVEF was evaluated by out-of-sample explained variance. Calibration was not formally assessed because the exploratory model did not show discrimination above chance. The study is reported following the reporting principles of the TRIPOD+AI statement, recognizing that the analysis is exploratory and not a prediction model [15]. Analyses were performed using the R system version 4.3.2 [16].

## 3. Results

### 3.1. Demographics and Baseline Echocardiography

We included 60 patients; 42 underwent mitral valve repair (MVRe), while 18 underwent mitral valve replacement (MVR). Thirty-seven (62%) were male, mean age was 68 +/− 11.8 years. Median EuroSCORE II and STS Score were 1.5% (0.9–2.5) and 0.8% (0.3–2.7), respectively. Incidence of comorbidities was low and renal function was generally preserved. At baseline assessment, NYHA was II in 43 patients (72%) and III in 17 (28%) with median NT-proBNP of 256 (110–559) pg/mL. Most patients (36; 60%) had no previous hospital admission for HF, and guideline-directed medical therapy for HF was used in approximately 50% of patients. For complete data see Table 1. MR grade was severe in 54 (90%) and moderate in six (10%) with mean regurgitant volume of 73.2 +/− 26.4 mL and low transvalvular gradient. Resting LV function was preserved (LVEF 59.5 +/− 7.9%), with mildly increased LV end-diastolic and end-systolic volumes (146.5 +/− 48.4 mL) and 58 (44–70) mL. Tricuspid regurgitation was mild in 36 (60%) and moderate in 16 (26.7%), with preserved right ventricular function and close-to-normal pulmonary pressure. Complete 2D-TTE data are listed in Table 2.

### 3.2. Cardiopulmonary Exercise Test (CPET) and Exercise Stress Echocardiography

All patients correctly completed the CPET full protocol with a mean workload of 99.8 +/− 46.9 W. Mean VO_2_ was reduced both at rest and at peak with a concomitantly lower-than-normal ventilatory anaerobic threshold (VAT). The VE/VCO_2_ slope was slightly reduced (33 +/− 8.9) as well as the Oxygen Uptake Efficiency Slope (OUES). Complete CPET data are reported in Table 3.

During ESE, we observed an increase in the LVOT-VTI, stroke volume and cardiac output that, however, remained below the expected values at peak as shown in Table 3. Mean flow reserve was 37.5 +/− 25%, with 24 patients (44%) showing preserved flow reserve (>40%). LVEF increased from rest to peak with preserved contractility reserve in 39 patients (74%). We observed a significant increase in sPAP during exercise reaching 56.6 +/− 15.7 mmHg at peak, while TAPSE increased from 22.2 +/− 3.8 mm to 26.8 +/− 4.9 mm. The TAPSE/sPAP ratio was 0.6 +/− 0.2 at rest and 0.5 +/− 0.2 at peak. Complete ESE data are reported in Table 3.

### 3.3. Outcomes and FU

Four patients died within 30 days; of those, one died from cardiovascular causes. Mortality was numerically higher in patients who underwent MVR than in those who underwent MVRe, although the difference was not statistically significant (16.7% vs. 2.4%; *p* = 0.08, Fisher’s exact test). In the three patients who experienced non-cardiovascular death, the cause was sepsis in two cases while in one case respiratory failure due to acute pneumonia. At discharge, patients were all in NYHA class I–II (3.6% and 96.4%, respectively). Discharge echocardiography showed LVEF 55 +/− 6.8% and LVEDV 109 +/− 30.3 mL. Residual MR at discharge was mild in 20 patients (40.8%), moderate in one patient (2%) and severe in none, with a mean transvalvular gradient of 3 +/− 1.03 mmHg. At early follow-up at 6.4 months (IQR 5.9–6.8), no additional deaths were observed. NYHA class was I in 15 patients (38.5%) and II in 24 (61.5%). No patient developed severe MR recurrence; mild MR was present in 25 patients (64.1%) and moderate MR in one (2.6%). The mean transvalvular gradient remained low (3 +/− 1.1 mmHg). LVEF was 55 +/− 8.3% and LVEDV was 107 +/− 25.3 mL. Right ventricular function was mildly reduced in 13 patients (33.3%), TAPSE was 17.5 (14.8–19.0) mm and sPAP was 28 +/− 3.7 mmHg. Complete data are shown in Table 4 and Table 5.

### 3.4. Exploratory Analysis of Outcome Predictors

**Figure 1:**
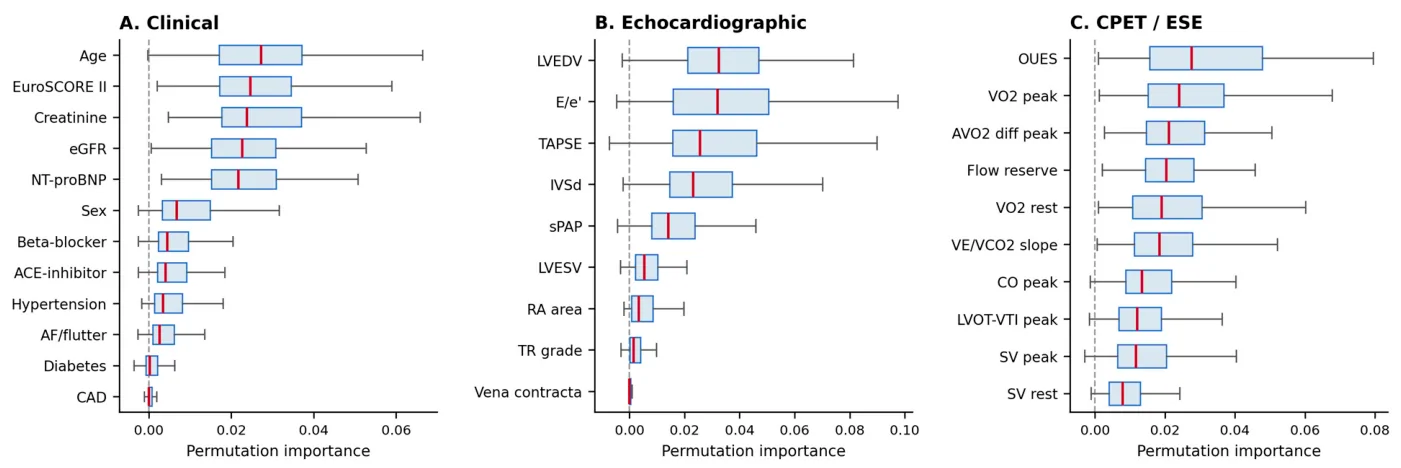
Exploratory random forest variable-importance distributions for NYHA-class improvement (bootstrap distribution, B = 200; classification forest) across baseline clinical (**A**), echocardiographic (**B**), and CPET/ESE (**C**) variables. The wide and overlapping bootstrap distributions, frequently crossing zero, indicate unstable rankings; therefore, individual variables are not interpreted as reliable predictors. eGFR: Estimated Glomerular Filtration Rate (mL/min); IVSd: Interventricular Septum Thickness (mm); LVEDV: Left Ventricle End-Diastolic Volume (mL); TAPSE: Tricuspid Annular Plane Systolic Excursion (mm); LVEDD: Left Ventricle End-Diastolic Diameter (mm); AR: Aortic Regurgitation; LVESD: Left Ventricle End-Systolic Diameter (mm); EROA: Effective Regurgitant Orifice Area (qcm); MR grade: Mitral Regurgitation grade; PWd: Left Ventricular Posterior Wall Thickness at End-Diastole (mm); sPAP: Systolic Pulmonary Arterial Pressure (mmHg); LVESV: Left Ventricular End-Systolic Volume (mL); AS: Aortic Stenosis; TR_grade: Tricuspid Regurgitation grade; RV: Right Ventricle function; MAC: Mitral Annular Calcification; LVEF: Left Ventricle Ejection Fraction (%); RA_area: Right Atrium area (qcm); VO_2__rest: Oxygen Uptake (mL/kg/min) at rest; VO_2__peak: Oxygen Uptake (mL/kg/min) at peak; VE/VCO_2__sl: Minute Ventilation/Carbon Dioxide Production Slope; OUES: Oxygen Uptake Efficiency Slope; AVO_2__diff: Arteriovenous Oxygen Difference (mL/dL); VO_2_/work_s: Oxygen Uptake/Work slope; PETCO_2_: partial pressure of end-tidal CO_2_ (mmHg); SpO_2__peak: Oxygen Saturation (%) at peak.

**Figure 2:**
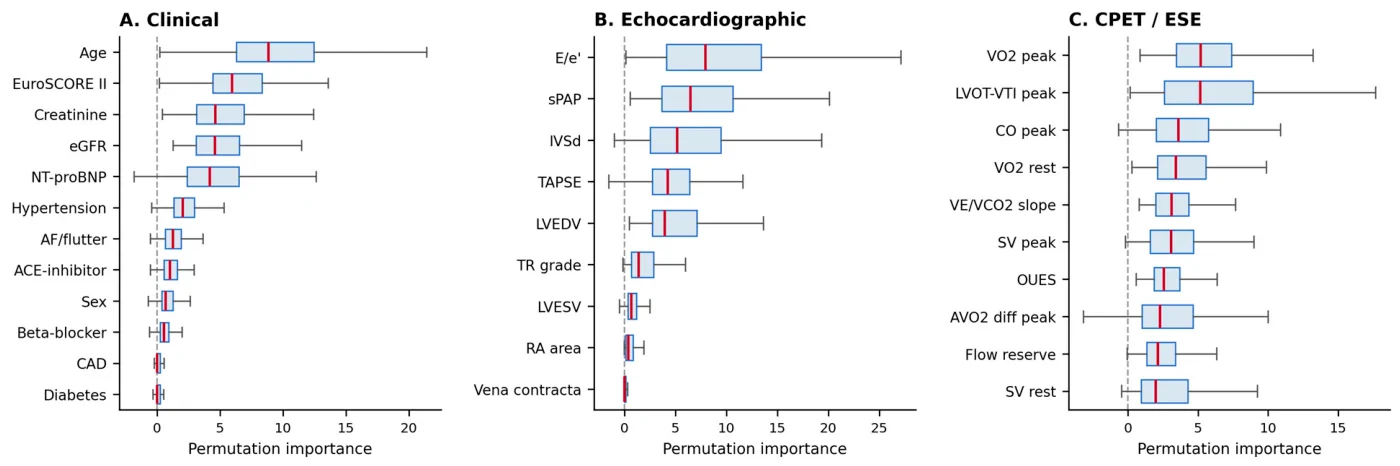
Exploratory random forest variable-importance distributions for the change in LVEF (bootstrap distribution, B = 200; regression forest) across baseline clinical (**A**), echocardiographic (**B**), and CPET/ESE (**C**) variables. The wide and overlapping bootstrap distributions indicate unstable rankings; therefore, individual variables are not interpreted as reliable predictors. eGFR: Estimated Glomerular Filtration Rate (mL/min); RA_area: Right Atrium area (qcm); TR_grade: Tricuspid Regurgitation grade; MAC: Mitral Annular Calcification; LVEDD: Left Ventricle End-Diastolic Diameter (mm); AR: Aortic Regurgitation; EROA: Effective Regurgitant Orifice Area (qcm); PWd: Left Ventricular Posterior Wall Thickness at End-Diastole (mm); TAPSE: Tricuspid Annular Plane Systolic Excursion (mm); RV: Right Ventricle function; IVSd: Interventricular Septum Thickness (mm); LVESV: Left Ventricular End-Systolic Volume (mL); sPAP: Systolic Pulmonary Arterial Pressure (mmHg); LVESD: Left Ventricle End-Systolic Diameter (mm); LVEF: Left Ventricle Ejection Fraction (%); MR_grade: Mitral Regurgitation grade; LVEDV: Left Ventricle End-Diastolic Volume (mL); AS: Aortic Stenosis; LVOT-VTI_rest_ESE: Left Ventricle Outflow Tract-Velocity Time Integral (cm) ESE at rest; SV_rest_ESE_pre: Stroke Volume (mL) during ESE at rest; SV_peak_cpet_pre: Stroke Volume (mL) during ESE at peak; WMSI_peak_ESE_pre: Wall Motion Score Index during ESE at peak; CO_peak_ESE_pre: Cardiac Output (L/min) during ESE at peak; EAT_ESE_pre: Epicardial Adipose Tissue during ESE (mm); LVOT_diameter_ESE_pre: Left Ventricle Outflow Tract Diameter (cm) during ESE; WMSI_rest_ESE_pre: Wall Motion Score Index during ESE at rest.

For both outcomes, the smaller outcome group per candidate predictor was small (NYHA improvement: 19–20 patients per outcome group for 9–12 predictors, approximately 1.6–2.1 events per variable; Delta LVEF: 32 patients for 9–11 predictors), well below the size required for reliable multivariable modeling. At this sample size, discrimination and calibration could not be reliably estimated: out-of-bag discrimination for NYHA improvement did not exceed chance in any predictor domain, the models explained essentially no out-of-sample variance in Delta LVEF, and bootstrap resampling showed largely unstable variable-importance rankings. Accordingly, the variable-importance orderings shown in Figure 1 and Figure 2 are exploratory and hypothesis-generating rather than a validated prediction model.

In an exploratory comparison, none of the baseline clinical, CPET or ESE parameters differed significantly between patients who did and those who did not improve by at least one NYHA class (all *p* > 0.15; Mann–Whitney U or Fisher’s exact test), consistent with the absence of a discriminative signal at this sample size. The change in LVEF at early follow-up was negligible (median 0, mean 0.2 +/− 5.8%), leaving essentially no variability to model. Of the 60 operated patients, 21 (35%) did not have a standardized early follow-up assessment for the functional outcome; patients with and without follow-up were comparable at baseline (age, EuroSCORE II, NYHA class and peak VO_2_, all *p* > 0.18), with a non-significant trend toward higher baseline LVEF among those with follow-up (63% vs. 58%, *p* = 0.07).

As a secondary exploratory analysis, a random forest approach, potentially more sensitive to non-linear effects and interactions, was concordantly non-discriminating: out-of-bag discrimination did not exceed chance and variable-importance rankings were unstable on bootstrap resampling. In Figure 1 and Figure 2, the wide and overlapping bootstrap distributions, frequently crossing zero, illustrate this instability; individual variable ranks are therefore not interpreted as reliable predictors.

## 4. Discussion

The main findings of the present study are that (1) patients who undergo surgical treatment of DMR present reduced functional capacity and impaired response to exercise despite mild or no symptoms and preserved LVEF; (2) CPET and ESE can be used safely and efficiently as part of a multiparametric evaluation of patients affected by DMR to characterize their functional and hemodynamic status after surgical treatment; and (3) in this exploratory analysis, the random forest models did not discriminate above chance, and variable-importance rankings were unstable.

Usually, surgery is indicated for patients affected by DMR on the base of symptoms or LV impairment [1,2]; however, these elements might underestimate the overall functional condition of this population. In our exploratory analysis, baseline LVEF did not provide a reliable discriminative signal for either NYHA-class improvement or early ΔLVEF. CPET is an important tool to evaluate functional capacity and has been used in the setting of heart failure (HF) [17,18,19,20,21] to estimate mortality outcomes. It has been demonstrated that patients affected by HF experienced negative outcomes when showing peak VO_2_ ≤ 14 mL/Kg/min, reduced VAT and an augmented VE/VCO_2_ slope. Our population presented mild symptoms and preserved LV function with moderately low levels of NT-proBNP. Despite this, their CPET performance showed objective functional impairment, with reduced peak VO_2_ and a VE/VCO_2_ slope above the commonly used threshold of 30 [12]. Similarly, ESE suggested an impaired hemodynamic response to exercise, with LVOT-VTI and stroke volume values in the lower range. Interestingly, only 44% of patients presented preserved flow reserve at ESE. This is consistent with the established role of CPET in assessing patients’ functional capacity. Messika-Zeitoun et al. previously demonstrated that reduced functional capacity assessed with CPET [22] predicts clinical events in asymptomatic patients with DMR. Moreover, functional capacity was influenced by the severity of MR and diastolic dysfunction. Impaired functional capacity, reflected by reduced peak VO_2_ and an elevated VE/VCO_2_ slope, is a plausible correlate of poorer functional recovery, consistent with Messika-Zeitoun’s findings. Gordon et al. [23] found that in patients with HF, OUES was associated with an increased risk of adverse events independently from the VE/VCO_2_ slope. However, in that study, the prognostic utility of OUES was inferior to peak VO_2_ [23]. There is limited evidence supporting the prognostic role of Arteriovenous Oxygen Difference in patients with DMR. This parameter may reflect peripheral oxygen extraction and is physiologically related to stroke volume and cardiac output. In MR, LVOT-VTI is usually reduced in favor of an increase in mitral valve (MV)-VTI since the flow through the MV increases with a simultaneous reduction in the systemic flow through the LVOT [24]. Scalia et al. found that the MV-VTI/LVOT-VTI ratio has a prognostic value at one year in patients treated with transcatheter edge-to-edge MV repair [24]. In the present study, we did not measure MV-VTI, so we were not able to calculate this ratio; this parameter may deserve evaluation in future studies. SV is directly correlated with LVOT-VTI and contributes to the physiological interpretation of forward-flow response. We acknowledge that our population was largely asymptomatic with low NT-proBNP levels and is not comparable with HF patients; however, it is interesting that this still compensated patients, which shows some similitudes with more advanced MV disease conditions, suggesting that we are probably missing the real functional decline of individuals affected by DMR as previously hypothesized by other authors [4,5,6,22].

Regarding baseline variables, medical treatment, including ACE inhibitors and beta-blockers, remains clinically relevant but cannot be interpreted as an independent predictor of either outcome in this exploratory cohort. This is consistent with previous studies, showing that GDMT can improve outcomes in patients with HF [25,26]. Diastolic dysfunction and elevated LV filling pressures are recognized contributors to reduced exercise tolerance in Mitral Regurgitation. This finding is in line with what was previously reported by Messika-Zeitoun et al. [22], who showed that diastolic dysfunction negatively affected functional capacity in patients with asymptomatic DMR as well as LVOT-VTI.

Because the exploratory rankings were unstable and the model did not discriminate above chance, individual variable ranks are not interpreted; the suggested associations require confirmation in larger, prospectively validated cohorts. From a physiological standpoint, a larger vena contracta may correspond to lower LVOT-VTI and reduced forward systemic flow.

## 5. Limitations

This study has important limitations. (1) It is a retrospective, single-center analysis of 60 patients, of whom 39 and 32 were evaluable for the functional and echocardiographic outcomes; with only ≈1.6–2.1 events per candidate predictor, the sample is well below the size required to develop or validate a multivariable clinical prediction model [1,2], and any multivariable model is at high risk of overfitting. Consistent with this, out-of-bag discrimination for the functional outcome did not exceed chance and the variable-importance rankings were unstable on resampling. We therefore do not present these analyses as a clinical prediction tool: the reported associations are exploratory and hypothesis-generating and require confirmation, with external validation and calibration, in larger multicenter prospectively collected cohorts. (2) Considering the retrospective nature of the study, postoperative functional evaluation relied only on NYHA class grading that might be imprecise in correct patient evaluation; moreover, NYHA class changes were assessed at 6 months—that could be too short of a period to exactly evaluate the effects of surgery. Prospective studies with longer follow-up and based on more precise functional status evaluation, as the 6 min walking test for example, are desirable to further support the preliminary findings of this study. (3) The use of LVEF changes to assess modifications in postoperative myocardial function in DMR can be misleading since LVEF is intrinsically flawed by the presence of the regurgitant fraction and underestimates real LV function; further prospective studies using more sophisticated and overload-independent parameters as correctedLVEF and forwardLVEF [9,27] are necessary to deeply understand the effect of surgery on LV function changes and its relation with functional capacity.

## 6. Conclusions

Patients undergoing surgical correction of DMR showed reduced functional capacity and abnormal haemodynamic response to exercise despite mild symptoms and preserved LVEF. CPET and ESE may therefore provide useful complementary information for preoperative functional and haemodynamic characterization. However, in this small retrospective cohort, exploratory random forest analyses did not show discrimination above chance and variable-importance rankings were unstable. These findings should be considered hypothesis-generating and require confirmation in larger, prospectively collected multicenter cohorts before predictive or decision-support applications can be proposed.

## Figures and Tables

**Table 1:** Baseline demographic and clinical characteristics.

	Mean ± SD, Median (I–III Quartile), N (%)
Age (years)	68 ± 11.8
Male	37 (62)
STS Score	0.8 (0.3–2.7)
EuroSCORE II	1.5 (0.9–2.5)
Creatinine (mg/dL)	0.9 (0.8–1.1)
eGFR (mL/min/1.73 m^2^)	74.2 ± 25
Diabetes Mellitus Type II	10 (17)
Arterial Hypertension	33 (55)
Coronary Artery Disease	4 (6.67)
Previous Myocardial Infarction	3 (5)
Previous PCI	4 (6.7)
Previous Stroke	0 (0)
COPD	5 (8.3)
Atrial Fibrillation/Flutter	21 (35)
ICD/Pacemaker	3 (5)
NYHA - II - III	43 (72) 17 (28)
NT-proBNP (pg/mL)	256 (110–559)
Antiplatelet Therapy	9 (15)
Beta-Blocker	32 (53.3)
ACE-Inhibitor	24 (40)
ARB	17 (28.3)
ARNI	1 (1.7)
MRA	18 (30)
SGLT2i	3 (5)
Diuretics	32 (53.3)

eGFR: Estimated Glomerular Filtration Rate; PCI: Percutaneous Coronary Intervention; COPD: Chronic Obstructive Pulmonary Disease; ICD: Implantable Cardioverter-Defibrillator; NYHA class: New York Heart Association Functional Classification; NT-proBNP: N-terminal Prohormone of Brain Natriuretic Peptide; ACE-Inhibitor: Angiotensin-converting-enzyme Inhibitors; ARB: Angiotensin II Receptor Blockers; ARNI: Angiotensin II Receptor Blockers and Neprilysin Inhibitor; MRA: Mineralocorticoid Receptor Antagonist; SGLT2i: Sodium-Glucose Cotransporter-2 inhibitors; Continuous variables are reported as mean ± SD (approximately symmetric distributions) or median (I–III quartile) (skewed distributions); categorical variables as N (%).

**Table 2:** Baseline 2D-transthoracic echocardiography characteristics.

	Mean ± SD, Median (I–III Quartile), N (%)
Mitral Regurgitation - Moderate–Severe - Severe	6 (10) 54 (90)
EROA (cm^2^)	0.4 (0.4–0.6)
Vena Contracta (mm)	7.4 ± 0.7
Regurgitant Volume (mL)	73.2 ± 26.4
Mean Mitral Valve Gradient (mmHg)	2 (1–3)
E/e’	12 (9.9–15.2)
E/A	1.5 (1.3–2.1)
LVEDD (mm)	57 ± 6.7
LVESD (mm)	37.2 ± 6.8
LVEDV (mL)	146.5 ± 48.4
LVESV (mL)	58 (44–70)
Interventricular Septum (diastole, mm)	11 ± 1.6
Posterior Wall (diastole, mm)	9.9 ± 1.2
LVEF (%)	59.5 ± 7.9
Left Atrial Volume (mL)	84.0 (74.5–98.0)
Right Atrium area (cmq)	18 (16–21)
Aortic Stenosis - Mild - Moderate	0 (0) 1 (1.7)
Aortic Regurgitation - Mild - Moderate	12 (20) 6 (10)
Right Ventricular Function - Mildly Reduced - Moderately Reduced - Severely Reduced	3 (5) 0 (0) 1 (1.7)
TAPSE (mm)	22.1 ± 3.9
sPAP (mmHg)	32 (26–47)
Tricuspid Regurgitation - Mild - Moderate	36 (60) 16 (26.7)

EROA: Effective Regurgitant Orifice Area; LVEDD: Left Ventricle End-Diastolic Diameter; LVESD: Left ventricle end systolic diameter; LVEDV: Left Ventricle End-Diastolic Volume; LVESV: Left ventricle end systolic volume; TAPSE: Tricuspid Annular Plane Systolic Excursion; sPAP: Systolic Pulmonary Arterial Pressure.

**Table 3:** Preoperative ESE and CPET.

	Mean ± SD, Median (I–III Quartile), N (%)
LVOT-VTI at rest (cm)	18.3 ± 3.9
Stroke volume at rest (mL)	51.0 (43.0–60.0)
Cardiac output at rest (L/min)	3.8 (3.2–5.1)
LVOT-VTI at 4 min (cm)	21.3 ± 3.8
Stroke volume at 4 min (mL)	62.2 ± 18.8
Cardiac output at 4 min (L/min)	5.9 ± 2.1
LVOT-VTI at anaerobic threshold (cm)	23.7 ± 3.9
Stroke volume at anaerobic threshold (mL)	69.3 ± 18.9
Cardiac output at anaerobic threshold (L/min)	7.7 ± 2.8
LVOT-VTI at peak (cm)	24.5 ± 4.9
Stroke volume at peak (mL)	72.5 ± 21.6
Cardiac output at peak (L/min)	9.0 ± 3.2
Flow Reserve (%)	37.5 ± 25
Flow Reserve	24 (44)
End Diastolic diameter (cm)	5.7 ± 0.8
End Systolic Diameter (cm)	3.6 (3.3–4.1)
End Diastolic diameter indexed (cm/m^2^)	3.0 (2.8–3.3)
End Systolic Diameter indexed (cm/m^2^)	2.0 (1.8–2.2)
Interventricular Septum (cm)	1.1 ± 0.1
Posterior Wall (cm)	0.9 ± 0.2
Left Ventricular Mass (g)	237.1 ± 73.7
Left Ventricular Mass indexed (g/m^2^)	127.3 ± 33.1
End Diastolic Volume at rest (mL)	166.3 ± 52.5
End Diastolic Volume indexed at rest (mL/m^2^)	89.7 (71.5–99.9)
End Diastolic Volume at 4 min (mL)	167.5 ± 53
End Diastolic Volume at AT (mL)	167.5 ± 53
End Diastolic Volume at peak (mL)	166.9 ± 52.7
End Systolic Volume at rest (mL)	54.0 (44.0–74.2)
End Systolic Volume indexed at rest (mL/m^2^)	29.2 (25.0–38.0)
End Systolic Volume at 4 min (mL)	48.0 (40.0–66.0)
End Systolic Volume at anaerobic threshold (mL)	46.5 (37.0–61.5)
End Systolic Volume at peak (mL)	44.0 (35.0–60.0)
End Systolic Volume indexed at peak (mL/m^2^)	24.9 (20.0–30.4)
LVEF at rest (%)	62 ± 10.8
LVEF at 4 min (%)	66.3 ± 9.7
LVEF at anaerobic threshold (%)	70.4 (64.5–74.8)
LVEF at peak (%)	72.7 (64.4–76.9)
ESE ΔLVEF, peak-rest (%)	7.8 (7.2–8.2)
Contractility Reserve (ΔEF > 7.5%)	39 (74)
Left Atrial Volume (mL)	88.0 (70.0–107.2)
Left Atrial Volume indexed (mL/m^2^)	48.5 (38.0–61.4)
Mean E/e’ at rest	11.6 (9.9–16.7)
Mean E/e’ at peak	11.0 (9.1–14.1)
Right Atrium area (cm^2^)	19.0 (16.0–24.0)
sPAP at rest (mmHg)	41.5 ± 14
sPAP at peak (mmHg)	56.6 ± 15.7
mPAP/Cardiac Output slope	1.7 (0.9–3.3)
TAPSE at rest (mm)	22.2 ± 3.8
TAPSE at peak (mm)	26.8 ± 4.9
TAPSE/sPAP at rest (mm/mmHg)	0.6 ± 0.2
TAPSE/sPAP at peak (mm/mmHg)	0.5 ± 0.2
Cardiopulmonary exercise test	
	Mean ± SD, Median (I–III quartile), N (%)
Workload (W)	99.8 ± 46.9
Time of Effort (min)	10 (9–11)
VO_2_ at rest (mL/kg/min)	6 ± 1.5
VO_2_ at 4 min (mL/kg/min)	10.1 ± 2.3
VO_2_ at anaerobic threshold (mL/kg/min)	14.9 ± 4.3
VO_2_ at anaerobic threshold % peak VO_2_	83.1 ± 10.2
VO_2_ at peak (mL/kg/min)	17.2 ± 5
VO_2_ at peak (% predicted)	75.1 ± 16.1
O_2_ pulse at peak (mL/beat)	10.1 ± 3.4
O_2_ pulse at peak (% predicted max)	96.7 ± 21.3
VE/VCO_2_ slope	33 ± 8.9
Breathing Reserve (%)	41.8 ± 14.3
SpO_2_ at rest (%)	98.0 (98.0–99.0)
SpO_2_ at peak (%)	98.0 (98.0–99.0)
Pet CO_2_ at rest (mmHg)	32.5 ± 4.2
Pet CO_2_ at peak (mmHg)	34.6 ± 5.4
OUES	1.6 (1.3–1.9)
AV O_2_ diff at rest (mL/dL)	11.3 ± 4.1
AV O_2_ diff at peak (mL/dL)	14.8 ± 4.2
AV O_2_ diff at peak/Hb (mL/gr)	1.1 ± 0.3

LVOT: Left Ventricle Outflow Tract; VTI: Velocity Time Integral; LVEF: Left Ventricular Ejection Fraction; sPAP: Systolic Pulmonary Arterial Pressure; mPAP: Median Pulmonary Arterial Pressure; TAPSE: Tricuspid Annular Plane Systolic Excursion; VO_2_: Oxygen Uptake; VE/VCO_2_ slope: Minute Ventilation/CO_2_ Output slope; SpO_2_: Oxygen Saturation; Pet CO_2_: End-Tidal Carbon Dioxide; OUES: Oxygen Uptake Efficiency Slope; AV O_2_ diff: Arteriovenous Difference; Hb: Hemoglobin.

**Table 4:** Postoperative data.

Demographics	Mean ± SD, Median (I–III Quartile), N (%)
NYHA class at discharge - I - II	2 (3.6) 54 (96.4)
All-Cause Death	4 (6.7)
Cardiovascular Death	1 (1.7)
Periprocedural Death	0 (0)
Surgical Revision for Bleeding	1 (1.7)
Neurological Events	0 (0)
AMI	0 (0)
AKI Increase VS Baseline > 150%	0 (0)
CVVH	0 (0)
Chronic Dialysis	0 (0)
Transient Conduction Disturbance	1 (1.7)
New Onset AF - Paroxysmal - Persistent	11 (18.3) 4 (6.7)
Pericardial Effusion - Minor - Major	3 (5) 1 (1.7)
Pleural Effusion - Minor - Major	13 (21.7) 5 (8.3)
Wound Dehiscence	2 (3.3)
Sepsis	4 (6.7)
Hospitalization (from surgery date) (days)	7 (6–9)
Discharge modality - Home - Death - Rehabilitation or Other Unit - Other ICU	26 (43.3) 4 (6.7) 30 (50) 0 (0)
Antiplatelet Agent	46 (82.1)
Beta-Blocker	38 (67.9)
ACE-inhibitor	26 (46.4)
ARB	4 (7.1)
ARNI	1 (1.8)
MRA	35 (62.5)
SGLT2i	3 (5.4)
Diuretics	44 (78.6)
Mitral Regurgitation - None/absent - Mild - Moderate - Severe	28 (57.1) 20 (40.8) 1 (2.0) 0 (0)
Mean Mitral Valve Gradient (mmHg)	3 ± 1.03
E/e’	13 ± 4.2
Left Ventricle End-Diastolic Volume (mL)	109 ± 30.3
LVEF (%)	55 ± 6.8
Left Atrial Volume (mL)	69 ± 18.9
Right Ventricle Dysfunction - Mild - Moderate - Severe	19 (38.8) 0 (0) 1 (2)
TAPSE (mm)	16 ± 2.9
sPAP (mmHg)	28.0 (26.0–35.0)
Tricuspid Regurgitation - Mild - Moderate - Severe	39 (79.6) 5 (10.2) 0 (0)

AMI: Myocardial Infarction Attack; AKI: Acute Kidney Injury; CVVH: Continuous Venovenous Hemofiltration; AF: Atrial Fibrillation; ACE-Inhibitor: Angiotensin-converting-enzyme Inhibitors; ARB: Angiotensin II Receptor Blockers; ARNI: Angiotensin II Receptor Blockers and Neprilysin Inhibitor; MRA: Mineralocorticoid Receptor Antagonist; SGLT2i: Sodium-Glucose Cotransporter-2 inhibitors; LVEF: Left Ventricle Ejection Fraction; TAPSE: Tricuspid Annular Plane Systolic Excursion; sPAP: Systolic Pulmonary Arterial Pressure.

**Table 5:** Follow-up data.

Demographics	Mean ± SD, Median (I–III Quartile), N (%)
All-Cause Death	0 (0)
Cardiovascular Death	0 (0)
Stroke	0 (0)
Myocardial Infarction	0 (0)
New-Onset AF	0 (0)
NYHA Class at Early Follow-up	
- I	15 (38.5)
- II	24 (61.5)
Hospitalization for HF	0 (0)
Re-intervention	0 (0)
Mitral Regurgitation - None/absent	13 (33.3)
- Mild	25 (64.1)
- Moderate	1 (2.6)
- Severe	(0)
Mean Mitral Valve gradient	3 ± 1.1
E/e’	11.4 ± 5.1
E/A	1 ± 0.3
LVEDV (mL)	107 ± 25.3
LVESV (mL)	52 ± 14.1
Interventricular Septum (diastole, mm)	11.0 (10.0–12.0)
Posterior Wall (diastole, mm)	9.9 ± 1.2
LVEF (%)	55 ± 8.3
Left Atrial Volume (mL)	55 ± 19.8
Right Ventricle Dysfunction - Mild - Moderate - Severe	13 (33.3) 0 (0) 0 (0)
TAPSE (mm)	17.5 (14.8–19.0)
sPAP (mmHg)	28 ± 3.7
Tricuspid Regurgitation - Mild - Moderate - Severe	30 (76.9) 4 (10.3) 1 (2.6)

AF: Atrial Fibrillation; NYHA class: New York Heart Association classification; HF: Heart Failure; LVEDV: Left Ventricle End-Diastolic Volume; LVESV: Left ventricle end systolic volume; LVEF: Left Ventricle Ejection Fraction; TAPSE: Tricuspid Annular Plane Systolic Excursion; sPAP: Systolic Pulmonary Arterial Pressure.

## Data Availability

The data presented in this study are available on request from the corresponding author due to privacy concerns.
